# Dr. R. J. Howe

**Published:** 1916-07

**Authors:** 


**Dr. R. J. Howe,** (not listed in Polk nor State Directory), died in Watkins, N. Y., Mch. 28.

